# Supplementary material for: The pharmacoepigenomic landscape of cancer cell lines reveals the epigenetic component of drug sensitivity
Source: Commun Biol. 2023 Aug 9;6:825. doi: 10.1038/s42003-023-05198-y (PMC10412573; doi:10.1038/s42003-023-05198-y)
Supplement: Supplementary file 2 — Supplementary Information [file 42003_2023_5198_MOESM2_ESM.pdf]

Supplementary information for

The pharmacoepigenomic landscape of cancer cell lines reveals the epigenetic component of drug sensitivity

Ohnmacht AJ, Rajamani A, Avar G, Kutkaite G, Gonçalves E, Saur D, Menden MP

Corresponding Menden MP.
E-mail: michael.menden@helmholtz-munich.de

This PDF file includes:

Supplementary Figure 1 to 7

Other supplementary information for this manuscript include:

Supplementary Data 1 to 3

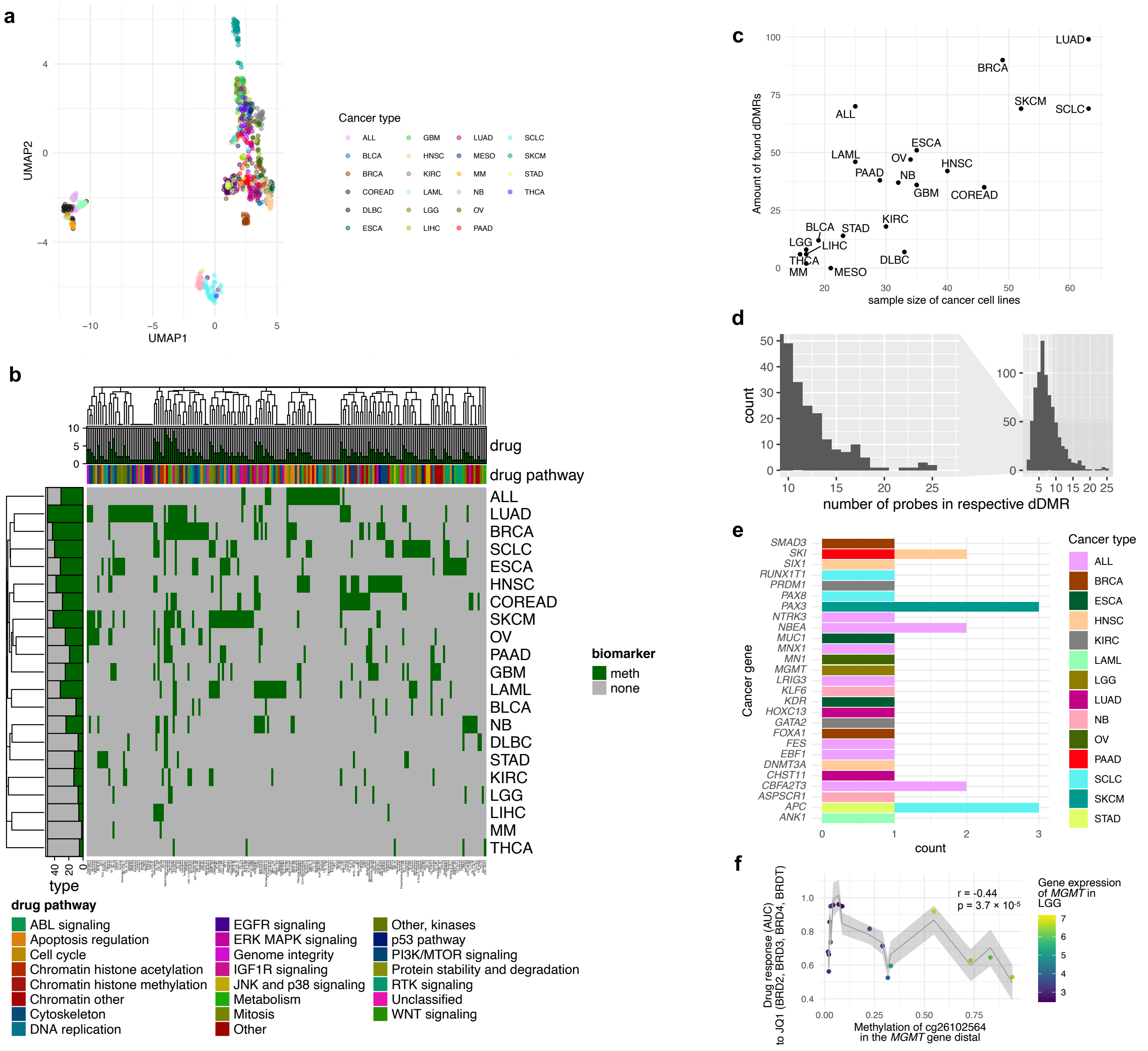

Supplementary Figure 1: Additional characterisations of dDMRs. (**a**) The UMAP dimensionality reduction for gene expression patterns of cell lines in GDSC. (**b**) A heatmap of drugs that showed at least one dDMR across the screened compounds and cancer types. (**c**) Scatter plot showing sample size of each cancer type against found dDMRs across all screened drugs. (**d**) A histogram of the number of sites in each dDMR. (**e**) Set of 27 dDMRs proximal to cancer genes. (**f**) dDMR in *MGMT* for response to JQ1 in low-grade glioma (LGG). The error bars corresponding to 95% confidence intervals, the raw p-value (p) for the respective CpG site and the Pearson correlation coefficient (r) are displayed.

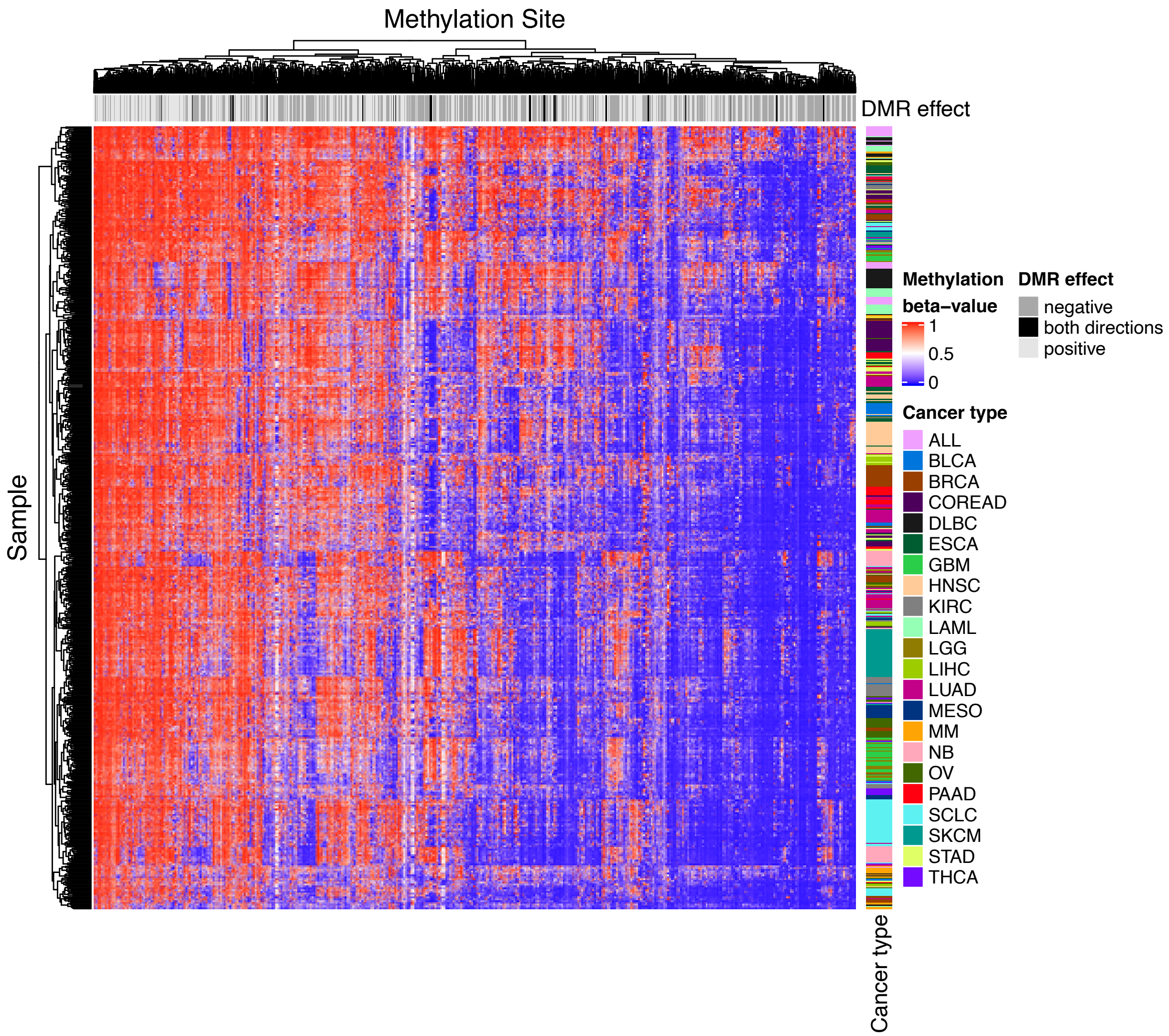

Supplementary Figure 2: Methylation pattern of dDMRs. Heatmap of dDMR methylation across cancer types.

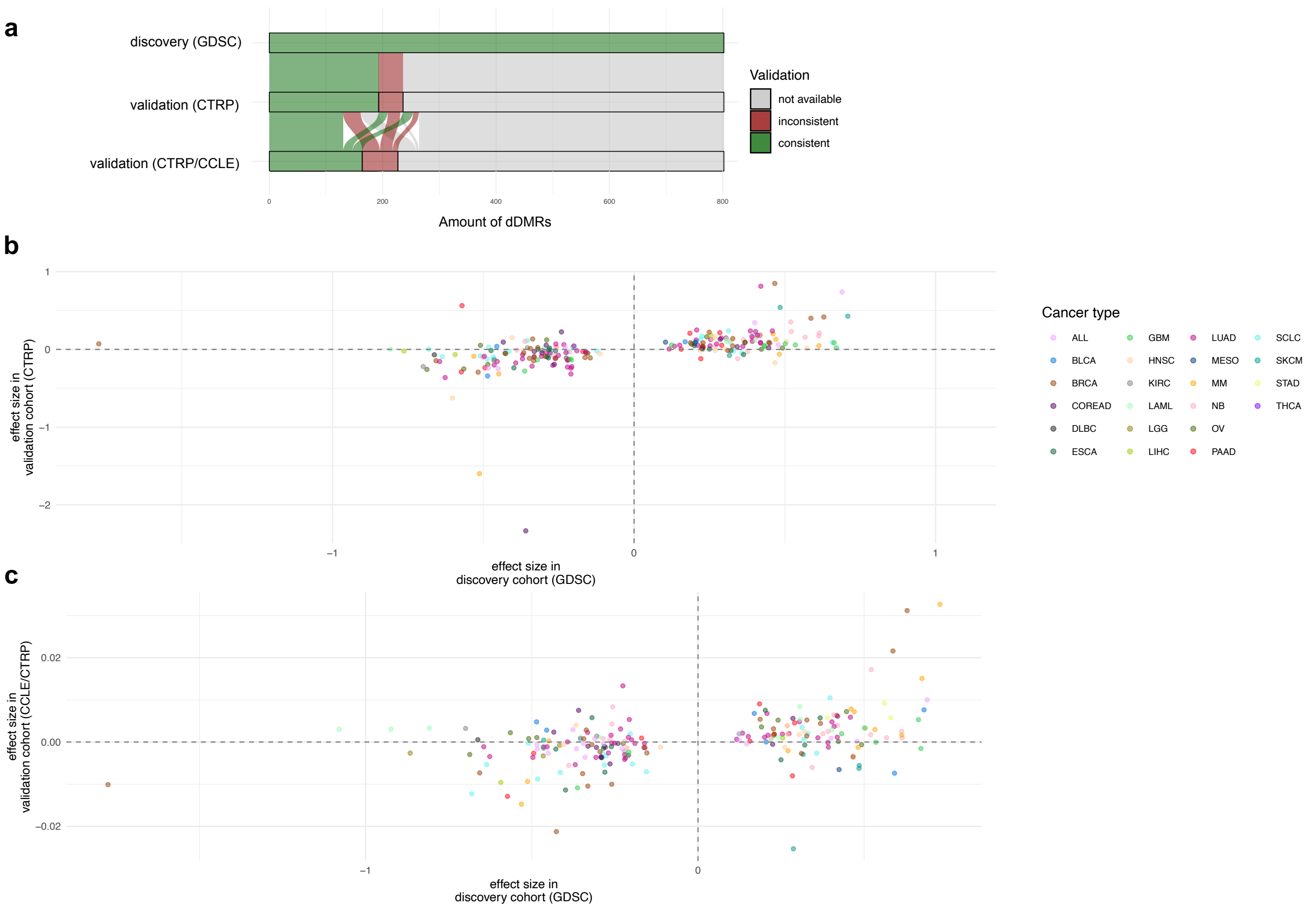

Supplementary Figure 3: Scatter plot of effect sizes from validation cohorts. (**a**) Consistency of effect sizes of 802 dDMRs from the GDSC discovery cohort validated in either the CTRP and CCLE datasets. (**b**) Effect sizes of dDMRs validated with the CTRP drug response HTS. (**c**) Effect sizes of overlapping dDMRs with the CCLE RRBS and CTRP data.

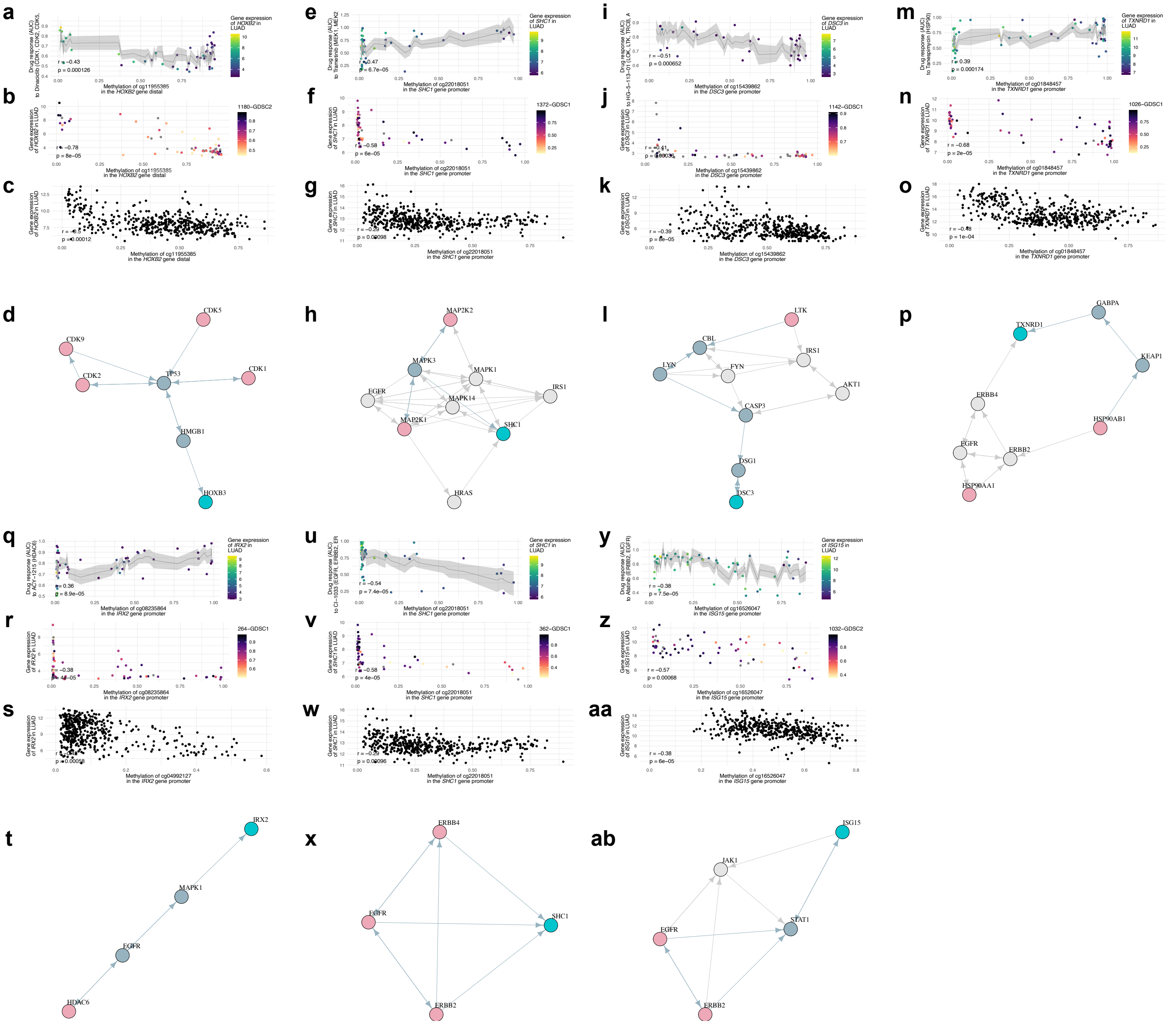

Supplementary Figure 4: tgdDMRs in LUAD. (**a**)-(**ab**) Correlation between drug response quantified by area-under-the-curve, DNA methylation and gene expression plus the corresponding protein-protein interaction network between putative drug target (pink) and tgdDMR-associated gene encoding protein (light blue). In the graph, nodes that are traversed with a shortest path are highlighted by the blue-grey colour among the alternative paths. For analysing DNA methylation and drug response, the error bars corresponding to 95% confidence intervals, the raw p-value (p) for each CpG site and the Pearson correlation coefficient (r) are reported. For analysing DNA methylation and gene expression, the empirical adjusted p-value (p) and the Pearson correlation coefficient (r) are reported.

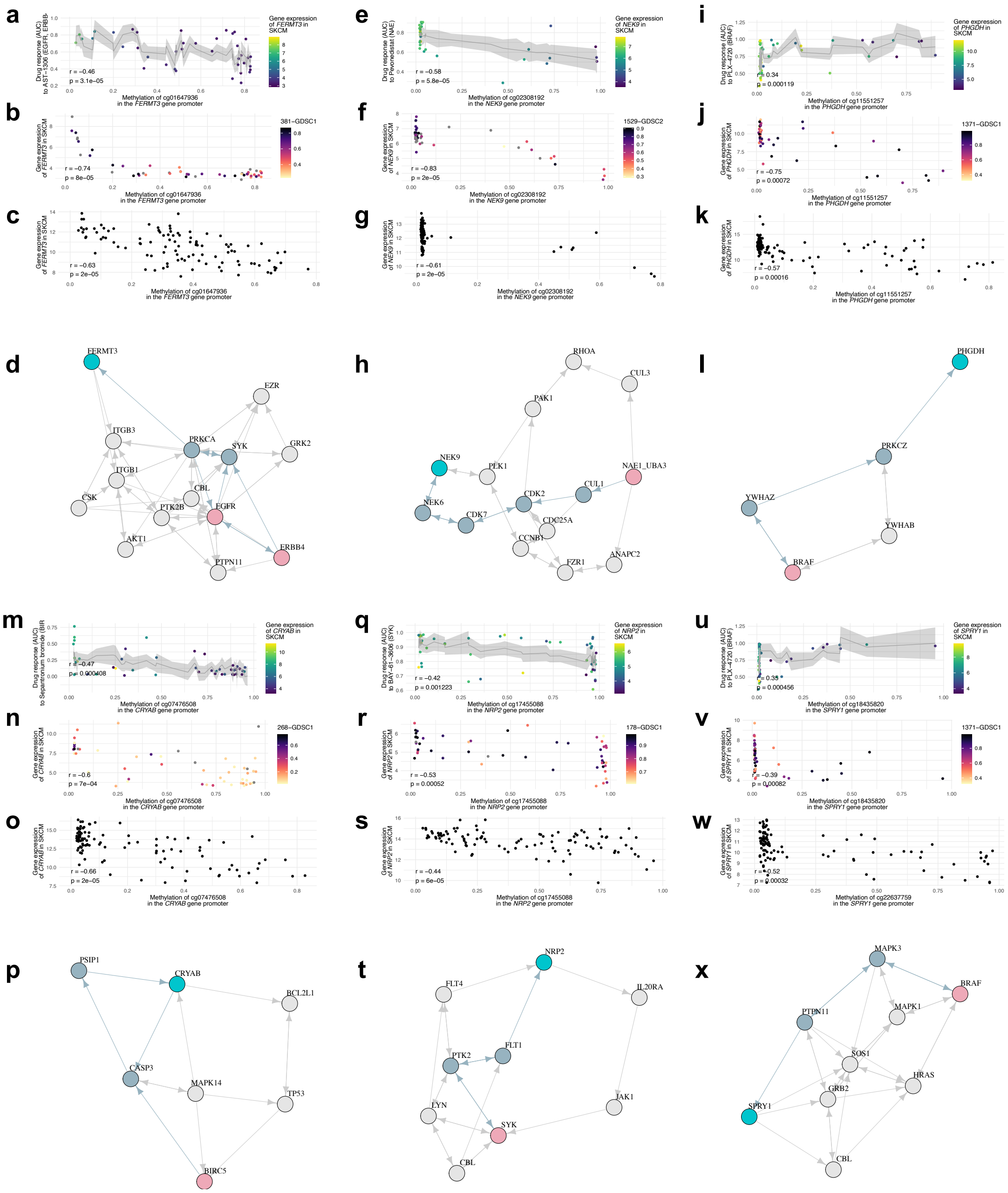

Supplementary Figure 5: tgdDMRs in SKCM. (**a**)-(**x**) Correlation between drug response quantified by area-under-the-curve, DNA methylation and gene expression plus the corresponding protein-protein interaction network between putative drug target (pink) and tgdDMR-associated gene encoding protein (light blue). In the graph, nodes that are traversed with a shortest path are highlighted by the blue-grey colour among the alternative paths. For analysing DNA methylation and drug response, the error bars corresponding to 95% confidence intervals, the raw p-value (p) for each CpG site and the Pearson correlation coefficient (r) are reported. For analysing DNA methylation and gene expression, the empirical adjusted p-value (p) and the Pearson correlation coefficient (r) are reported.

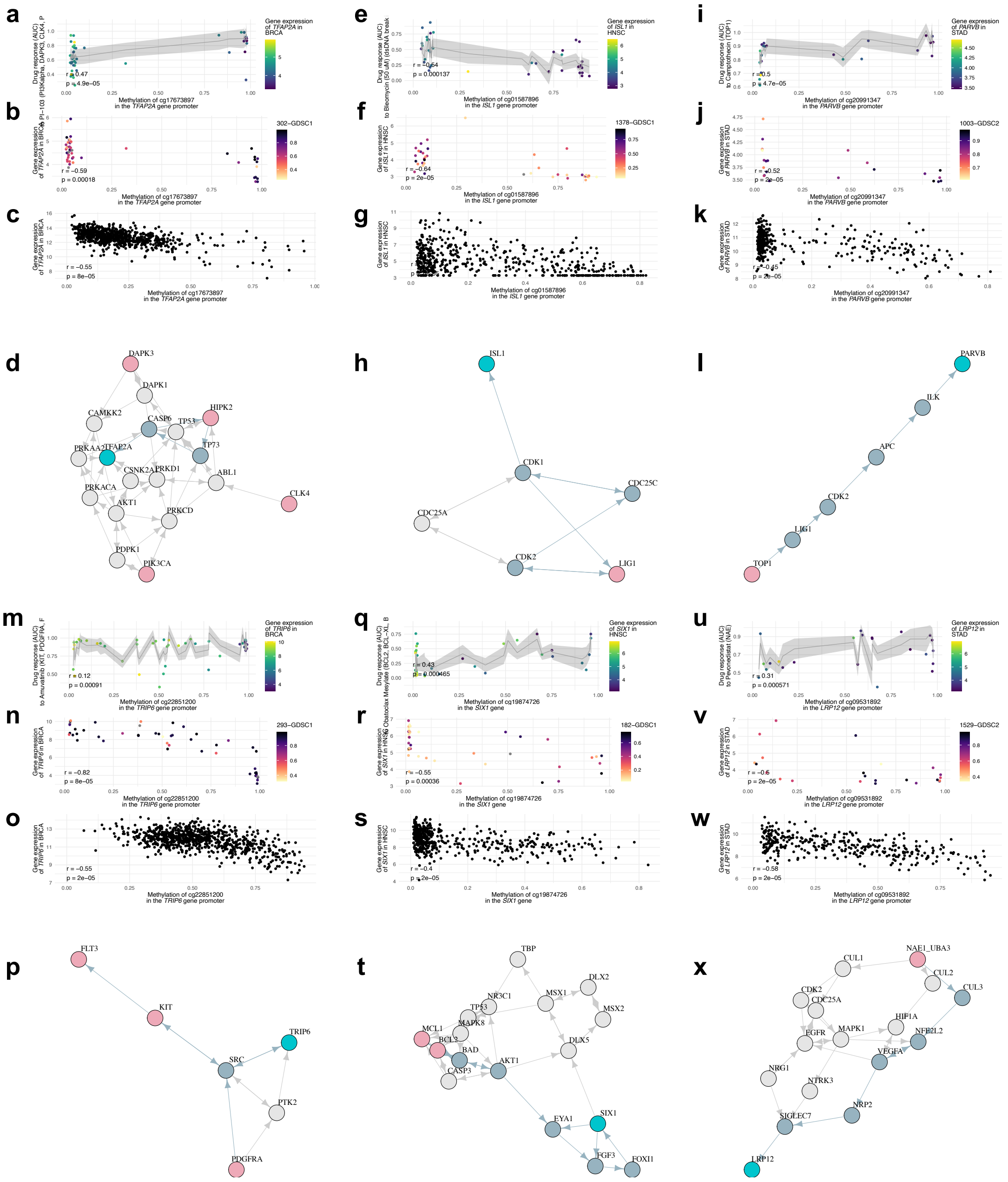

Supplementary Figure 6: tgdDMRs in BRCA, HNSC and STAD. (**a**)-(**x**) Correlation between drug response quantified by area-under-the-curve, DNA methylation and gene expression plus the corresponding protein-protein interaction network between putative drug target (pink) and tgdDMR-associated gene encoding protein (light blue). In the graph, nodes that are traversed with a shortest path are highlighted by the blue-grey colour among the alternative paths. For analysing DNA methylation and drug response, the error bars corresponding to 95% confidence intervals, the raw p-value (p) for each CpG site and the Pearson correlation coefficient (r) are reported. For analysing DNA methylation and gene expression, the empirical adjusted p-value (p) and the Pearson correlation coefficient (r) are reported.

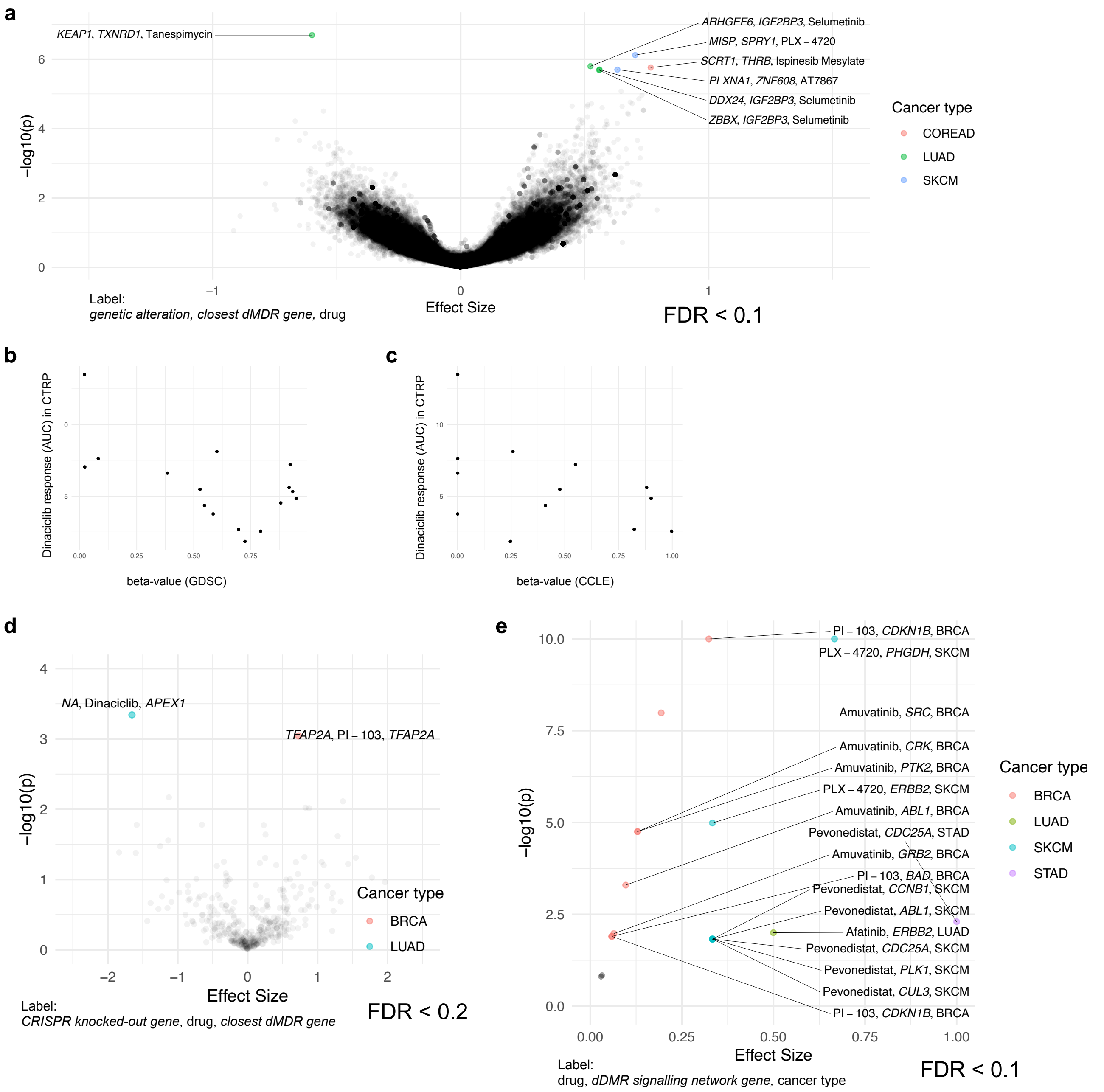

Supplementary Figure 7: tgdDMRs in the context of genetic alterations, CRISPR screens and drug signatures. (**a**) A volcano plot summarising associations between tgdDMRs and somatic mutations in cancer cell lines. (**b**,**c**) Scatter plot for drug response to dinaciclib (AUC) in the CTRP validation set with the HumanMethylation450 BeadChip array in GDSC and reduced representation bisulfite sequencing in the CCLE independent dataset. (**d**) A volcano plot summarising associations between tgdDMRs and CRISPR knockout screens of the genes associated with tgdDMRs and their signalling network neighbourhood. (**e**) A volcano plot summarising enrichments of genes associated with tgdDMRs and their signalling network neighbourhood in the LINCS drug signatures for the matching compound and cancer type.
