# Supplementary material for: The pharmacoepigenomic landscape of cancer cell lines reveals the epigenetic component of drug sensitivity
Source: Commun Biol. 2023 Aug 9;6:825. doi: 10.1038/s42003-023-05198-y (PMC10412573; doi:10.1038/s42003-023-05198-y)
Supplement: Supplementary file 3 — Description of Additional Supplementary Files [file 42003_2023_5198_MOESM3_ESM.pdf]

Description of Additional Supplementary Files

File Name: Supplementary Data 1
Description: Enrichment of drug classes in dDMRs. Summary statistics of hypergeometric tests f or drug class enrichments. The table contains drug class annotations, the associated hypergeometric test p-value testing for either enrichment or depletion, the adjusted p-value using the Benjamini-Hochberg false discovery rate correction, the tested cancer type and the sample size.

File Name: Supplementary Data 2
Description: 802 dDMRs, 377 short-listed dDMRs, 58 tgdDMRs and 19 tgdDMRs with proteinprotein interaction networks. Summary statistics and annotations of identified dDMRs and tgdDMRs, annotated by drug and its putative drug target, the cancer type, the genomic region, its functional region and exact position, the adjusted p-value from the discovery cohort, the tested sample size, the proximal associated gene in cancer cell lines and primary tumours, the adjacent gene, the nature of the correlation between the methylation and expression in both cancer cell lines and primary tumours, the associated derived mechanism, the association p-value between the proximal gene expression and drug response, the number of sites in the respective region, information about a potential DHS, enhancer, an associated signalling network, the cancer gene information, its validation status in the CTRP and CCLE cohorts and associations of the their methylation with genetic alterations, CRISPR knockout screens and LINCS drug signatures.

File Name: Supplementary Data 3
Description: The source data for the figures in the manuscript.
