# Supplementary material for: The pharmacoepigenomic landscape of cancer cell lines reveals the epigenetic component of drug sensitivity
Source: Commun Biol. 2023 Aug 9;6:825. doi: 10.1038/s42003-023-05198-y (PMC10412573; doi:10.1038/s42003-023-05198-y)
Supplement: Supplementary file 7 — Reporting Summary [file 42003_2023_5198_MOESM7_ESM.pdf]

nature portfolio

Corresponding author(s): M. P. Menden

Last updated by author(s): Jul 31, 2023

Reporting Summary

Nature Portfolio wishes to improve the reproducibility of the work that we publish. This form provides structure for consistency and transparency in reporting. For further information on Nature Portfolio policies, see our Editorial Policies and the Editorial Policy Checklist.

Statistics

For all statistical analyses, confirm that the following items are present in the figure legend, table legend, main text, or Methods section.

n/a	Confirmed	
☐	☒	The exact sample size (*n*) for each experimental group/condition, given as a discrete number and unit of measurement
☐	☒	A statement on whether measurements were taken from distinct samples or whether the same sample was measured repeatedly
☐	☒	The statistical test(s) used AND whether they are one- or two-sided<br>*Only common tests should be described solely by name; describe more complex techniques in the Methods section.*
☐	☒	A description of all covariates tested
☐	☒	A description of any assumptions or corrections, such as tests of normality and adjustment for multiple comparisons
☐	☒	A full description of the statistical parameters including central tendency (e.g. means) or other basic estimates (e.g. regression coefficient) AND variation (e.g. standard deviation) or associated estimates of uncertainty (e.g. confidence intervals)
☐	☒	For null hypothesis testing, the test statistic (e.g. *F*, *t*, *r*) with confidence intervals, effect sizes, degrees of freedom and *P* value noted<br>*Give P values as exact values whenever suitable.*
☒	☐	For Bayesian analysis, information on the choice of priors and Markov chain Monte Carlo settings
☐	☒	For hierarchical and complex designs, identification of the appropriate level for tests and full reporting of outcomes
☐	☒	Estimates of effect sizes (e.g. Cohen's *d*, Pearson's *r*), indicating how they were calculated

Our web collection on statistics for biologists contains articles on many of the points above.

Software and code

Policy information about availability of computer code

Data collection	The raw methylation profiling data from GDSC, generated with the Infinium HumanMethylation450 BeadChip array, were downloaded from the Gene Expression Omnibus (GEO: accession number GSE68379 https://www.ncbi.nlm.nih.gov/geo/query/acc.cgi?acc=GSE68379). The raw methylation profiling data from CCLE, generated with the reduced representation bisulfite sequencing (RRBS) methylation profiling technology, were downloaded the fastq files of the RRBS data from the Sequence Read Archive (SRA: accession number PRJNA523380 https://www.ncbi.nlm.nih.gov/bioproject/PRJNA523380/) using the SRA toolkit. For the human primary tumours in TCGA, the preprocessed beta-values from the Infinium HumanMethylation450 BeadChip were downloaded from the GDC data portal (https://portal.gdc.cancer.gov/), accessed on the 18th October 2019. They were downloaded with the R package TCGAbiolinks. For the cell lines in the GDSC project, we downloaded the RMA-processed Affymetrix array data from their website http://www.cancerrxgene.org/gdsc1000/, accessed on the 8th August 2019. For the human tumours, we downloaded the Hi-Seq count data from the RNAseq experiments in the TCGA database https://portal.gdc.cancer.gov/, accessed on the 18th October 2019. For the discovery cohort, we leveraged HTS from the GDSC project http://www.cancerrxgene.org/downloads/bulk_download release 8.0. For the independent validation cohort, we used the Cancer Therapeutics Response Portal (CTRP) project https://portals.broadinstitute.org/ctrp.v2.1. The GDSC project has compiled a selection of somatic variants and copy number alterations 11, which are available at Cell Model Passports (https://cellmodelpassports.sanger.ac.uk/downloads). CRISPR knockout data and associated gene effects on viability were downloaded from the DepMap Public 22Q4 primary files (https://depmap.org/portal/download/all/). We used the CLUE knowledge base (https://clue.io/lincs) 74 and its provided API to retrieve transcriptomic gene signatures from the overlapping compounds with matching tissue.

Data analysis

The source code for the presented analysis is available at https://github.com/MendenLab/pheb. It refers to a runnable docker image that contains all used software for data analysis. The statistical analysis can be reproduced with the source code and datasets provided in Zenodo 75.

For manuscripts utilizing custom algorithms or software that are central to the research but not yet described in published literature, software must be made available to editors and reviewers. We strongly encourage code deposition in a community repository (e.g. GitHub). See the Nature Portfolio guidelines for submitting code & software for further information.

Data

Policy information about availability of data

All manuscripts must include a data availability statement. This statement should provide the following information, where applicable:

- Accession codes, unique identifiers, or web links for publicly available datasets
- A description of any restrictions on data availability
- For clinical datasets or third party data, please ensure that the statement adheres to our policy

All datasets that were analysed in this study are publicly available within the outlined repositories. Specifically, the GDSC and CCLE DNA methylation data is available on Gene Expression Omnibus (GEO: accession number GSE68379 https://www.ncbi.nlm.nih.gov/geo/query/acc.cgi?acc=GSE68379) and Sequence Read Archive (SRA: accession number PRJNA523380 https://www.ncbi.nlm.nih.gov/bioproject/PRJNA523380/), respectively. The TCGA DNA methylation data is available on the GDC data portal https://portal.gdc.cancer.gov/. The GDSC and CCLE drug response data are available on http://www.cancerrxgene.org/downloads/bulk_download release 8.0 and the Cancer Therapeutics Response Portal https://portals.broadinstitute.org/ctrp.v2.1, respectively. The GDSC and TCGA gene expression data is available on http://www.cancerrxgene.org/gdsc1000/ and the GDC data portal https://portal.gdc.cancer.gov/, respectively. The GDSC somatic variants and copy number alterations are available at Cell Model Passports https://cellmodelpassports.sanger.ac.uk/downloads. The CRISPR screens are available on DepMap https://depmap.org/portal/download/all/ and the LINCS data is available on CLUE https://clue.io/lincs. The processed datasets are publicly available on Zenodo 75. Source data for the figure panels are provided in Supplementary Data 3.

Research involving human participants, their data, or biological material

Policy information about studies with human participants or human data. See also policy information about sex, gender (identity/presentation), and sexual orientation and race, ethnicity and racism.

Reporting on sex and gender	Gender was not collected and analysed in the presented analysis. Probes for methylation profiling falling on sex chromosomes were excluded from the analysis in order to avoid sex biases.
Reporting on race, ethnicity, or other socially relevant groupings	A previous report on ethnic disparities in the TCGA reported that in the analysed tumour types participants were 77% white, 12% black, 3% Asian, 3% Hispanic and other ethnic groups with <0.5% (10.1001/jamaoncol.2016.1854). Additional demographic information of TCGA primary tumours are available under https://portal.gdc.cancer.gov/ and have been reported previously (10.1038/s41416-018-0140-8).
Population characteristics	Cancer cell lines and primary tumours from different cancer types were included but analysed separately. Demographic information is not applicable for cancer cell lines and demographic information of TCGA primary tumours are available under https://portal.gdc.cancer.gov/.
Recruitment	Potential biases include an enrichment in samples from European descent (10.1001/jamaoncol.2016.1854).
Ethics oversight	Ethics and policies regarding the TCGA study are available at https://www.cancer.gov/about-nci/organization/ccg/research/structural-genomics/tcga/history/policies.

Note that full information on the approval of the study protocol must also be provided in the manuscript.

Field-specific reporting

Please select the one below that is the best fit for your research. If you are not sure, read the appropriate sections before making your selection.

☒ Life sciences ☐ Behavioural & social sciences ☐ Ecological, evolutionary & environmental sciences

For a reference copy of the document with all sections, see nature.com/documents/nr-reporting-summary-flat.pdf

Life sciences study design

All studies must disclose on these points even when the disclosure is negative.

Sample size	The sample sizes of the GDSC, CCLE/CTRP and TCGA data were predetermined by their data availability.
Data exclusions	We selected cancer types with > 15 distinct molecularly characterised cell lines in the GDSC dataset. For the matching cancer types, all distinct primary tumour samples with both available DNA methylation and gene expression data in the CCLE and TCGA data were selected.
Replication	The reproducibility of response biomarkers was assessed by the CCLE/CTRP methylation and drug response data as independent validation cohort. Discrepancies between drug response biomarkers in CCLE/CTRP may arise due to technical noise or differences in drug screening assays, but showed high consistency as reported. The statistical analysis can be reproduced with the source code and datasets provided in

Zenodo 75.

Randomization	Cancer cell lines in the GDSC were treated with cancer compounds in a parallel fashion with the previously published study protocol.
Blinding	Blinding is not performed in this retrospective and exploratory study.

Reporting for specific materials, systems and methods

We require information from authors about some types of materials, experimental systems and methods used in many studies. Here, indicate whether each material, system or method listed is relevant to your study. If you are not sure if a list item applies to your research, read the appropriate section before selecting a response.

Materials & experimental systems

n/a	Involved in the study
☒	☐ Antibodies
☒	☐ Eukaryotic cell lines
☒	☐ Palaeontology and archaeology
☒	☐ Animals and other organisms
☒	☐ Clinical data
☒	☐ Dual use research of concern
☒	☐ Plants

Methods

n/a	Involved in the study
☒	☐ ChIP-seq
☒	☐ Flow cytometry
☒	☐ MRI-based neuroimaging
